# Combined transcriptome and metabolome analysis reveals nitrogen-responsive genes and metabolites in sugar beet (*Beta vulgaris* L.) seedlings

**DOI:** 10.3389/fpls.2026.1912820

**Published:** 2026-09-21

**Authors:** Zhi Li, Xianguo Ren, Yongkang Zhang, Xiaoxia Guo, Kang Han, Lu Tian, Yinghao Li, Huimin Ren, Yongjian Kang, Zixing Zhu, Wenbin Su, Chunyan Huang, Caiyuan Jian

**Affiliations:** Institute of Specialty Crops, Inner Mongolia Academy of Agricultural and Animal Husbandry Sciences, Hohhot, Inner Mongolia, China

**Keywords:** metabolomics, multi-omics co-enrichment, nitrogen metabolism, NRT2, sugar beet, transcriptomics

## Abstract

Nitrogen (N) is an essential macronutrient for the growth and yield of sugar beet (*Beta vulgaris* L.); however, the molecular mechanisms governing its responses to varying N supply levels have not been fully characterized. Sugar beet seedlings were cultivated under low nitrogen (LN, 0.35 g N kg^-1^) and high nitrogen (HN, 0.42 g N kg^-1^) conditions on the same nutrient-soil background. Leaf and root tissues were collected at 30 days after emergence (DAE) for RNA sequencing and untargeted metabolomics analysis. HN treatment increased plant height by 18.60%, fresh weight by 23.31%, and SPAD values by 27.98% at 35 DAE (*P* < 0.05). RNA-seq detected 1, 472 differentially expressed genes (DEGs) and 148 differentially accumulated metabolites (DAMs) in leaves, and 1, 224 DEGs and 261 DAMs in roots. KEGG co-enrichment analysis identified nitrogen metabolism, amino acid biosynthesis, and alanine-aspartate-glutamate metabolism as pathways jointly enriched by DEGs and DAMs. Fourteen nitrogen metabolism-related DEGs exhibited tissue-specific expression patterns: five DEGs encoding nitrogen assimilation enzymes (GLT1, NIA, and NIR) were upregulated in leaves under HN, participating in the stepwise conversion of nitrate to glutamate; eight DEGs predominantly encoding high-affinity nitrate transporters (NRT2.1 and NRT2.5) were uniformly downregulated in roots under HN. Pearson correlation analysis linked leaf nitrogen assimilation genes to O-phospho-L-serine and D-erythrose-4-phosphate (positive), whereas root *NRT2* family genes showed negative correlations with these metabolites. qRT-PCR results supported the expression patterns of key DEGs. Our results illustrate the coordinated transcriptome-metabolome responses in leaves and roots of sugar beet seedlings to different nitrogen levels and provide potential candidate genes and metabolites for future functional validation aimed at improving nitrogen use efficiency in sugar beet.

## 1 Introduction

Sugar beet (*Beta vulgaris* L.) is one of the most important sugar crops worldwide, serving as a primary feedstock for the sugar industry (Dohm et al., 2013; Jammer et al., 2020). Nitrogen (N) is a macronutrient central to plant growth, development, and productivity (Zayed et al., 2023). In sugar beet cultivation, nitrogen availability directly affects biomass accumulation, taproot yield, and sugar content (Zhang et al., 2017; Varga et al., 2021; Li et al., 2024). Although adequate nitrogen supply promotes vigorous vegetative growth and high sugar yields, excessive nitrogen application leads to resource waste, environmental pollution, and reduced beet quality (Jammer et al., 2020; Li et al., 2023a). A clearer picture of how sugar beet responds to varying nitrogen levels at the molecular level would help optimize fertilizer management and improve nitrogen use efficiency (NUE) (Govindasamy et al., 2023). In plants, nitrogen metabolism involves a series of coordinated processes, including nitrogen uptake, transport, reduction, and assimilation. Nitrate (NO_3_^-^) is the predominant form of inorganic nitrogen in aerobic soils and is absorbed by root cells through nitrate transporters (NRTs) (Krapp et al., 2014). Among these, members of the NRT2 family, as components of the high-affinity transport system, are particularly important under low nitrogen conditions (Perez-Delgado et al., 2016; Li et al., 2022; Deng et al., 2023). Once inside the cell, nitrate is first reduced to nitrite by nitrate reductase (NR), and subsequently further reduced to ammonium by nitrite reductase (NiR). Ammonium is then assimilated into organic nitrogen compounds through the glutamine synthetase/glutamate synthase (GS/GOGAT) cycle, generating glutamine and glutamate as the primary amino donors for downstream biosynthetic pathways (Perchlik and Tegeder, 2017; Chang et al., 2024). These processes are regulated at both the transcriptional and metabolic levels in response to nitrogen availability.

Transcriptomics and metabolomics, used together, can dissect crop responses to nutritional stress at complementary levels. Transcriptomics identifies differentially expressed genes (DEGs) (Zhang et al., 2017; Li et al., 2023a, 2022), and metabolomics captures downstream changes in metabolite pools that mirror physiological states more directly than transcript abundance alone (Xin et al., 2019a; Chang et al., 2024). Combining the two layers can expose regulatory connections that a single omics platform is likely to miss (Sun et al., 2023). This combined strategy has yielded detailed nitrogen-response maps in rice (Xin et al., 2019a, 2019b) and has been applied to study nutritional stress responses in other crops. In sugar beet, transcriptomics and metabolomics have been individually applied to investigate low nitrogen stress (Li et al., 2023a; Liu et al., 2020a) and salt stress (Liu et al., 2020b); However, multi-omics integrative analysis of nitrogen responses, particularly the coordinated transcriptomic and metabolomic reprogramming in leaf and root tissues, has not yet been conducted. Li et al. (2023a) dissected carbon-nitrogen balance under low nitrogen stress using transcriptomics alone, without examining metabolite-level responses; Liu et al. (2020a) focused on *NRT* gene family expression but did not integrate metabolomic data. This work differs from previous sugar beet nitrogen studies in scope. By combining transcriptome and metabolome across both leaves and roots, we were able to link tissue-specific gene regulation to metabolite accumulation through co-enrichment and correlation analyses. Here, we performed integrated transcriptomic and metabolomic analyses on leaves and roots of sugar beet seedlings grown under low nitrogen (LN, 0.35 g N kg^-1^) and high nitrogen (HN, 0.42 g N kg^-1^) conditions on the same nutrient-soil background, with the following objectives: (1) to characterize the phenotypic effects of different nitrogen levels on seedling growth; (2) to identify nitrogen-responsive DEGs and DAMs in leaves and roots; (3) to determine key nitrogen metabolism-related genes and metabolites through integrative pathway analysis; and (4) to reconstruct the molecular framework of nitrogen-responsive pathways in sugar beet seedlings.

## 2 Materials and methods

### 2.1 Plant materials and experimental treatments

The sugar beet monogerm pelleted seed cultivar KWS1197, produced by KWS (Germany), was used in this study. The pot experiment was conducted in 2024 at the Inner Mongolia Academy of Agricultural and Animal Husbandry Sciences (Hohhot, Inner Mongolia, China). The growth substrate consisted of vermiculite and nutrient soil mixed at a ratio of 1:3 (v/v). Before fertilizer addition, the background total nitrogen content of the substrate was measured total N, 0.30 g N kg^-1^. Therefore, the LN and HN treatments represent nitrogen addition levels on this same substrate background rather than nitrogen levels in an inert nitrogen-free medium. Based on previous nitrogen gradient experiments on sugar beet (Li et al., 2024) and related studies (Li et al., 2023a, b), two nitrogen treatments were established: LN, 0.35 g N kg^-1^ substrate total nitrogen and HN, 0.42 g N kg^-1^ substrate total nitrogen, representing lower and higher nitrogen supply conditions, respectively. 0.35 and 0.42 g N kg^-1^ was obtained by amending the nutrient substrate, that contained 0.30 gN kg^-1^, with 0.05 and 0.12 g N kg^-1^ of the urea (with a nitrogen content of 46%) fertilizer produced by Inner Mongolia Tianrun Fertilizer Co., Ltd. The total weight of each pot of substrate was 3 kg. Phosphorus (P) and potassium (K) fertilizers were uniformly applied at 0.10 g kg^-1^. All fertilizers (N, P, and K) were pre-mixed into the substrate as basal fertilizers before sowing. The experiment followed a completely randomized design with six biological replicates per treatment. Plastic pots (20 cm diameter, 14 cm height) were used, with 10 uniformly sized seeds sown per pot. All pots were placed in a controlled-environment growth chamber under the following conditions: temperature 25/18 °C (day/night), photoperiod 16/8 h (light/dark), and relative humidity 60%-70%, and intensity of illumination 300 μmol m^-2^ s^-1^. The pH and electrical conductivity (EC) of the substrate were measured before sowing (pH 7.8 ± 0.1; EC 0.46 ± 0.03 dS m^-1^). Plants were watered every 2–3 days with deionized water to maintain substrate moisture at approximately 60–70% of water-holding capacity, monitored gravimetrically by weighing pots. Pot positions within the growth chamber were re-randomized every 7 days to minimize positional effects from uneven light or airflow distribution. Ten seeds were sown per pot and thinned to five uniform seedlings per pot at 7 DAE to standardize plant density.

### 2.2 Growth parameter measurements

Plant growth parameters were measured at 15 and 35 days after emergence (DAE). Five uniformly growing plants were randomly selected from each replicate per treatment. Plant height and root length were measured using a tape measure (precision: 0.1 cm), plant height was measured from the leaf tip to the base of the shoot, and root length was measured from the root base to the tip of the longest root. Fresh weight per plant was determined using an electronic analytical balance (precision: 0.01 g). Leaf relative chlorophyll content (SPAD value) was measured at the tip of the third fully expanded leaf using a SPAD-502 chlorophyll meter (Konica Minolta, Japan). The average of five plants per replicate was calculated for statistical analysis. The morphometric measurements are destructive, so multiple replicates are required for each treatment.

### 2.3 Sampling for transcriptomics and metabolomics

Leaf and root tissues of sugar beet seedlings from both nitrogen treatments were collected at 30 DAE. This time point was selected to capture early transcriptional and metabolic events of nitrogen response rather than secondary effects of late-stage phenotypic outcomes. Samples were immediately flash-frozen in liquid nitrogen and stored at -80 °C until use. Four biological replicates per treatment per tissue were prepared for transcriptomic and metabolomic analyses, except HNVR1, which contained only three biological replicates, yielding a total of 15 samples: HNVL1 (HN leaves), LNVL1 (LN leaves), HNVR1 (HN roots), and LNVR1 (LN roots). Specifically, growth parameters measured at 15 DAE showed no significant differences between HN and LN treatments (**Table 1**), indicating that nitrogen-induced molecular changes had not yet translated into detectable phenotypic divergence at this early stage. By 35 DAE, significant differences in plant height, fresh weight, and SPAD values were established (**Table 1**), suggesting that by this late stage, secondary physiological and developmental consequences of prolonged nitrogen treatment would likely confound the primary transcriptional and metabolic signals of nitrogen response.

**Table 1:** Effects of different nitrogen levels on growth parameters of sugar beet seedlings.

DAE	Treatment	Plant height (cm)	Root length (cm)	Fresh weight (g)	SPAD
15	HN	17.77 ± 1.78 a	5.80 ± 0.52 a	17.00 ± 1.73 a	41.33 ± 0.83 a
15	LN	16.90 ± 1.85 a	5.63 ± 0.32 a	16.33 ± 2.08 a	40.77 ± 0.91 a
35	HN	34.00 ± 1.80 a	9.33 ± 1.15 a	134.00 ± 4.58 a	49.12 ± 1.82 a
35	LN	28.67 ± 1.53 b	8.67 ± 0.58 a	108.67 ± 3.51 b	38.38 ± 1.70 b

Values are mean ± SD (n = 6). Different lowercase letters within the same column at the same time point indicate significant differences at P < 0.05 (Duncan’s test). Statistical comparisons were made between LN and HN within each sampling time point. DAE, days after emergence; HN, high nitrogen; LN, low nitrogen.

### 2.4 RNA extraction, library construction, and sequencing

Total RNA was extracted from each sample using TRIzol reagent (Invitrogen, USA) according to the manufacturer’s instructions. RNA quality and integrity were assessed using 1% agarose gel electrophoresis and a NanoDrop 2000 spectrophotometer (Thermo Fisher Scientific, USA). RNA samples with OD 260/280 ratios between 1.8 and 2.1 and RNA integrity numbers (RIN) greater than 7.0 were selected for library construction. cDNA libraries were prepared using the NEBNext Ultra RNA Library Prep Kit for Illumina (New England Biolabs, USA) and sequenced on the Illumina NovaSeq 6000 platform to generate 150 bp paired-end reads.The sequencing company was Novogene, located in Tianjin, China. Raw reads were filtered using fastp (v0.23.2; Chen et al., 2018) to remove adapter sequences and low-quality reads. Clean reads were aligned to the sugar beet reference genome (IMA1, https://ngdc.cncb.ac.cn/gwh/Assembly/8860/show, GWHAMMD00000000; Dohm et al., 2013; McGrath et al., 2023) using HISAT2 (v2.2.1; Kim et al., 2019). Reads were assigned to genes using featureCounts (Liao et al., 2014) to generate raw count matrices for differential expression analysis. FPKM (fragments per kilobase of transcript per million mapped reads) values were calculated from these counts solely for expression visualization (e.g., heatmaps) and are not used for differential expression testing.

### 2.5 Identification of differentially expressed genes

Differential gene expression analysis between HN and LN treatments was performed using DESeq2 (Love et al., 2014) in R, which models raw count data with a negative binomial distribution and does not use FPKM values for statistical testing. Genes with |log_2_(fold change)| ≥ 1 and adjusted *P* (padj) ≤ 0.05 were defined as DEGs. To further focus on candidate genes with substantial expression changes and reduce false-positive rates, a more stringent threshold of |log_2_(fold change)| ≥ 3 (i.e., more than 8-fold expression difference) was applied in downstream functional analyses of nitrogen metabolism pathways, following screening strategies employed in previous crop multi-omics studies (Xin et al., 2019b). Kyoto Encyclopedia of Genes and Genomes (KEGG) pathway enrichment analyses of DEGs were performed using the clusterProfiler package (Wu et al., 2021) in R, with a significance threshold of padj < 0.05.

### 2.6 Untargeted metabolomics analysis

Untargeted metabolomics analysis of leaf and root samples was performed using liquid chromatography-tandem mass spectrometry (LC-MS/MS). Metabolite extraction, chromatographic separation, and mass spectrometric detection were conducted in both positive and negative ion modes. Metabolite extraction was performed as follows: freeze-dried leaf and root samples were ground to a fine powder using a mixer mill (MM 400, Retsch, Germany) with a zirconia bead at 30 Hz for 1.5 min. Approximately 100 mg of each sample was extracted overnight at 4 °C with 1.0 mL of 70% aqueous methanol containing 0.1 mg/L lidocaine as an internal standard. Following centrifugation at 10, 000 × g for 10 min, the supernatant was filtered through a 0.22 µm PTFE filter and transferred to an autosampler vial for LC-MS/MS analysis. Chromatographic separation was performed on an ultra-performance liquid chromatography (UPLC) system equipped with a C18 reverse-phase column (ACQUITY UPLC BEH C18, 1.7 µm, 2.1 × 100 mm, Waters, USA). The mobile phase consisted of (A) water containing 0.1% (v/v) formic acid and (B) acetonitrile containing 0.1% (v/v) formic acid. The elution gradient was programmed as follows: 0–1 min, 5% B; 1–11 min, 5–95% B; 11–12 min, 95% B; 12–12.1 min, 95–5% B; 12.1–15 min, 5% B. The flow rate was 0.35 mL/min, the column temperature was maintained at 40 °C, and the injection volume was 2 µL. Mass spectrometric detection was performed using a triple quadrupole time-of-flight mass spectrometer (AB SCIEX TripleTOF 5600+, SCIEX, USA) equipped with an electrospray ionization (ESI) source, operating in both positive and negative ion modes. The ESI parameters were as follows: ion spray voltage, 5.5 kV (positive mode) and −4.5 kV (negative mode); source temperature, 550 °C; curtain gas, 35 psi; ion source gas 1 (nebulizer), 50 psi; ion source gas 2 (heater), 50 psi. Mass spectra were acquired over the range of m/z 50–1, 200. Tandem mass spectrometry (MS/MS) spectra were acquired in information-dependent acquisition (IDA) mode, with a collision energy (CE) ramp of 30 ± 15 eV, a declustering potential of 80 V, and 10 candidate ions monitored per cycle.

Raw LC-MS/MS data were processed for feature detection, alignment, and normalization using Analyst TF 1.7.1 software (SCIEX, USA) coupled with the XCMS package (v3.4.0; Smith et al., 2006) in R for peak picking, retention time alignment, and peak grouping. The data preprocessing workflow included the following steps: (1) peak detection and extraction of mass spectral features; (2) chromatographic peak alignment across all samples to correct for retention time shifts; (3) missing value handling, where features detected in fewer than 50% of samples within a group were removed, and remaining missing values were imputed using half of the minimum detected value in the same group; (4) data normalization using the internal standard and total ion current (TIC) to correct for instrumental drift and ensure comparability across samples; and (5) quality control (QC) procedures, including the analysis of pooled QC samples at regular intervals throughout the acquisition sequence to monitor system stability. The tight clustering of QC samples near the center of the PCA score plots (**Figure 1**) confirmed the stability and reproducibility of the analytical system throughout the experiment.

**Figure 1:**
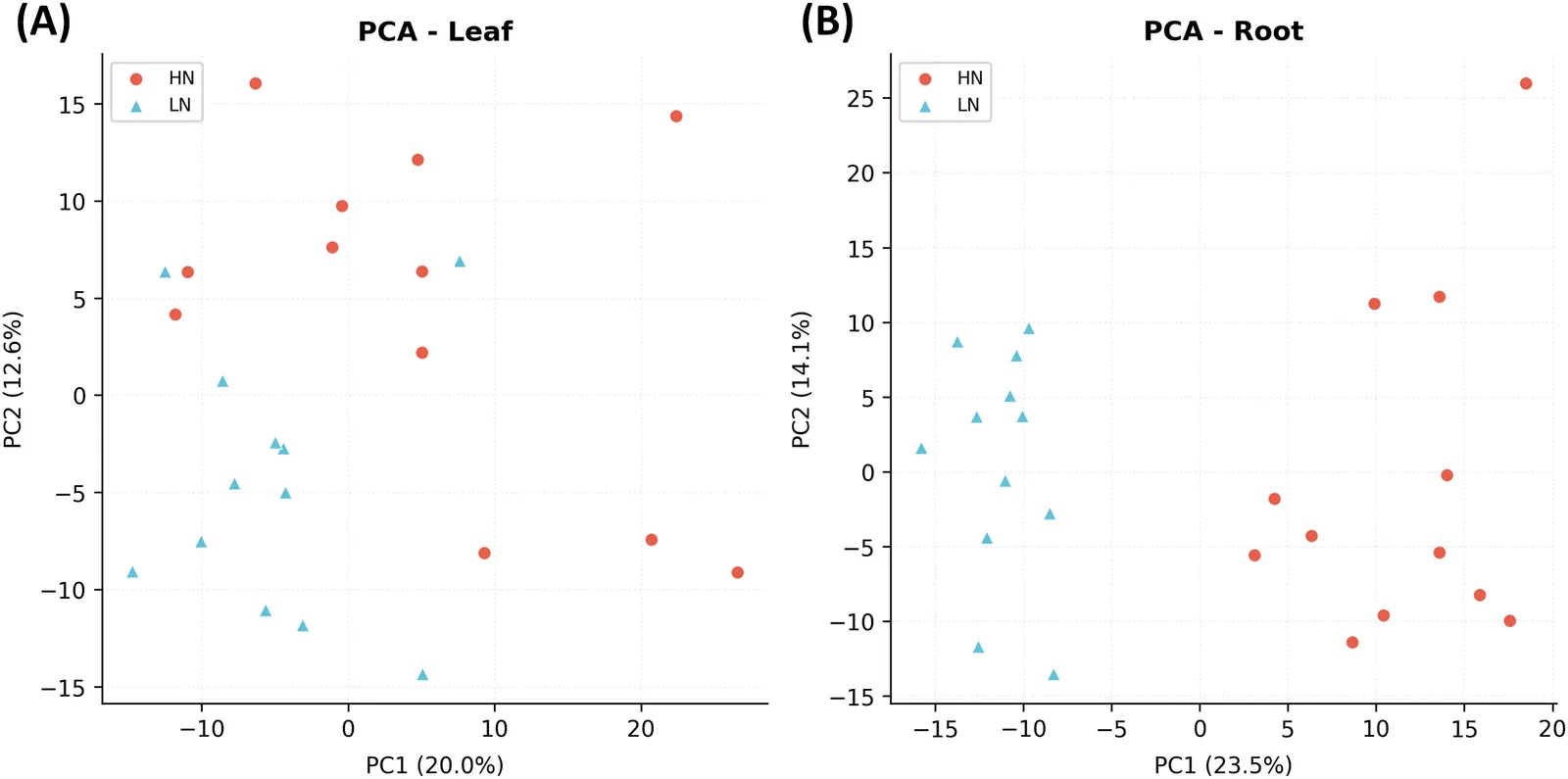
Principal component analysis (PCA) of metabolomic profiles of sugar beet tissues. **(A)** Leaf tissue showing separation between HN and LN groups. **(B)** Root tissue showing separation between HN and LN groups. Red circles: HN group; blue triangles: LN group.

Metabolite annotation was performed by matching acquired mass spectral data against the KEGG database (https://www.genome.jp/kegg/pathway.html), the Human Metabolome Database (HMDB; https://hmdb.ca/metabolites), and the LIPIDMaps database (http://www.lipidmaps.org/). Annotation was based on a combination of accurate mass matching (mass error < 30 ppm), MS/MS spectral similarity comparison with database entries and published reference spectra, and retention time information where available. According to the Metabolomics Standards Initiative (MSI) reporting standards (Sumner et al., 2007), the majority of metabolite annotations in this study correspond to MSI Level 2 (putatively annotated compounds based on physicochemical properties and/or spectral similarity to reference databases, without authentic standard confirmation) or Level 3 (putatively characterized compound classes). No metabolites were confirmed at MSI Level 1 (identified compounds) due to the absence of authentic standards. A total of 1556 mass spectral features were detected across all samples (combining positive- and negative-ion modes from both tissues), of which 409 metabolites were annotated and used for subsequent differential analysis. Of these annotated metabolites, 324 (79.2%) were classified as MSI Level 2.

DAMs were screened based on the following criteria: variable importance in projection (VIP) > 1.0, fold change (FC) > 1.2 or FC < 0.833, and *P* < 0.05 (Student’s t-test). We acknowledge that VIP scores, fold-change thresholds, and raw *P* values are all derived from the same underlying dataset and should not be regarded as three statistically independent safeguards against false-positive findings. Permutation testing of the OPLS-DA model evaluates the overall robustness of the supervised model but does not control the feature-level false discovery rate (FDR) arising from multiple univariate comparisons. To address this, we report both raw *P* values and Benjamini-Hochberg-adjusted *P* values (q-values) for all analyzed metabolites. The q-values are provided for every tested feature in both ionization modes and both tissues, allowing readers to evaluate significance at any FDR threshold. A sensitivity analysis was performed to determine how many DAMs remain significant at FDR < 0.05. Given the exploratory nature of this study and the limited sample size (*n* = 3 per group), the primary DAM set was defined using the nominal *P* < 0.05 threshold, and the metabolites selected by this criterion are described throughout as nominally significant candidate metabolites. The FDR-based sensitivity results are reported alongside the primary analysis to allow readers to assess the robustness of the findings. Prior to differential analysis, principal component analysis (PCA) was performed using the prcomp function in R to assess overall metabolic variation and evaluate biological replicate quality. Orthogonal partial least squares-discriminant analysis (OPLS-DA) was subsequently performed using MetaboAnalystR (Pang et al., 2021) to maximize inter-group separation and calculate VIP scores for each metabolite. The robustness of OPLS-DA models was evaluated through 200 permutation tests, and only models with Q² > 0.5 were considered reliable. KEGG pathway enrichment analysis was subsequently performed on the identified DAMs. Positive- and negative-ion mode data were processed and analyzed independently; DAMs identified in each mode were subsequently combined for pathway-level analyses. The total number of DAMs reported for each tissue represents the sum of DAMs from both ionization modes.

The thresholds for DAM identification were selected based on widely used criteria in plant metabolomics studies (Xin et al., 2019a; Liu et al., 2020b). The VIP > 1.0 threshold, derived from the OPLS-DA model, identifies metabolites with above-average contribution to inter-group separation, while the FC cutoff of 1.2 (equivalent to |log_2_FC| ≥ 0.263) ensures that only metabolites with biologically meaningful fold changes are retained. These metabolomics-specific thresholds differ from the |log_2_FC| ≥ 1 cutoff used for DEG identification because metabolite abundance changes are typically more subtle than transcript-level changes, and applying the same stringent fold-change threshold would exclude biologically relevant metabolic responses. Throughout this article, fold change values for metabolites are reported as FC (linear fold change), while gene expression changes are reported as log_2_FC, consistent with the conventions of each respective field. Because the primary DAM criterion uses nominal P < 0.05 without FDR correction, the biological interpretations based on these metabolites are presented as exploratory findings requiring validation in independent experiments with larger sample sizes.

### 2.7 Combined transcriptome and metabolome analysis

To identify co-regulated genes and metabolites, joint analysis of transcriptomic and metabolomic data was performed. DEGs and DAMs were mapped to shared KEGG metabolic pathways to identify co-enriched pathways. Pathways simultaneously containing significant DEGs and DAMs were regarded as key regulatory pathways in the nitrogen response of sugar beet. The identified DEGs and DAMs were mapped onto the nitrogen metabolism pathway to reconstruct and visualize coordinated transcriptomic and metabolic changes in leaves and roots. To quantify the regulatory relationships between gene expression and metabolite accumulation, Pearson correlation coefficients were calculated between the expression levels of nitrogen metabolism-related DEGs and the relative abundances of co-enriched DAMs using the cor.test function in R. Correlations with |r| > 0.8 and *P* < 0.05 were considered statistically significant, and correlation heatmaps were generated using the pheatmap package. The present analysis should be viewed as a first-pass, exploratory integration that identifies candidate gene-metabolite associations for hypothesis-driven follow-up.

### 2.8 qRT-PCR validation

To validate the RNA-seq results, quantitative real-time PCR (qRT-PCR) was performed on selected nitrogen metabolism-related DEGs. Total RNA was reverse-transcribed into cDNA using the PrimeScript RT Reagent Kit with gDNA Eraser (TaKaRa, Japan). qRT-PCR reactions were performed on a CFX96 Real-Time PCR Detection System (Bio-Rad, USA) using TB Green Premix Ex Taq II (TaKaRa, Japan). The sugar beet *BvActin* gene was used as the internal reference for normalization. Each reaction included three biological replicates and three technical replicates. Relative gene expression levels were calculated using the 2^-ΔΔCt^ method (Livak and Schmittgen, 2001).

### 2.9 Statistical analysis

Phenotypic data were analyzed separately at each sampling time point using one-way analysis of variance (ANOVA), followed by Duncan’s multiple range test at a significance level of *P* < 0.05. Statistical analyses were performed using SPSS 26.0 (IBM, USA). Graphs were generated using GraphPad Prism 8.0.1 (GraphPad Software, USA). Data are presented as mean ± standard deviation (SD). Different lowercase letters indicate statistically significant differences between treatments within the same sampling time point (*P* < 0.05). Throughout this article, raw *P* values are denoted as *P*, adjusted *P* values from DESeq2 are denoted as padj, and Benjamini-Hochberg-adjusted *P* values for metabolomics are denoted as FDR.

## 3 Results

### 3.1 Effects of different nitrogen levels on sugar beet seedling growth

The growth performance of sugar beet seedlings under the two nitrogen treatments is summarized in **Table 1**; **Figure 2**. At 15 DAE, no significant differences were observed between HN and LN treatments in plant height, root length, fresh weight per plant, or leaf SPAD values (P > 0.05). By 35 DAE, seedlings under HN treatment had greater plant height, fresh weight per plant, and leaf SPAD values than those under LN treatment (all *P* < 0.05), corresponding to increases of 18.60%, 23.31%, and 27.98%. Root length showed a similar trend but did not reach statistical significance (*P* > 0.05). Together, these data show that nitrogen supply promoted above-ground growth and chlorophyll accumulation, with the effects becoming detectable only at the later growth stage.

**Figure 2:**
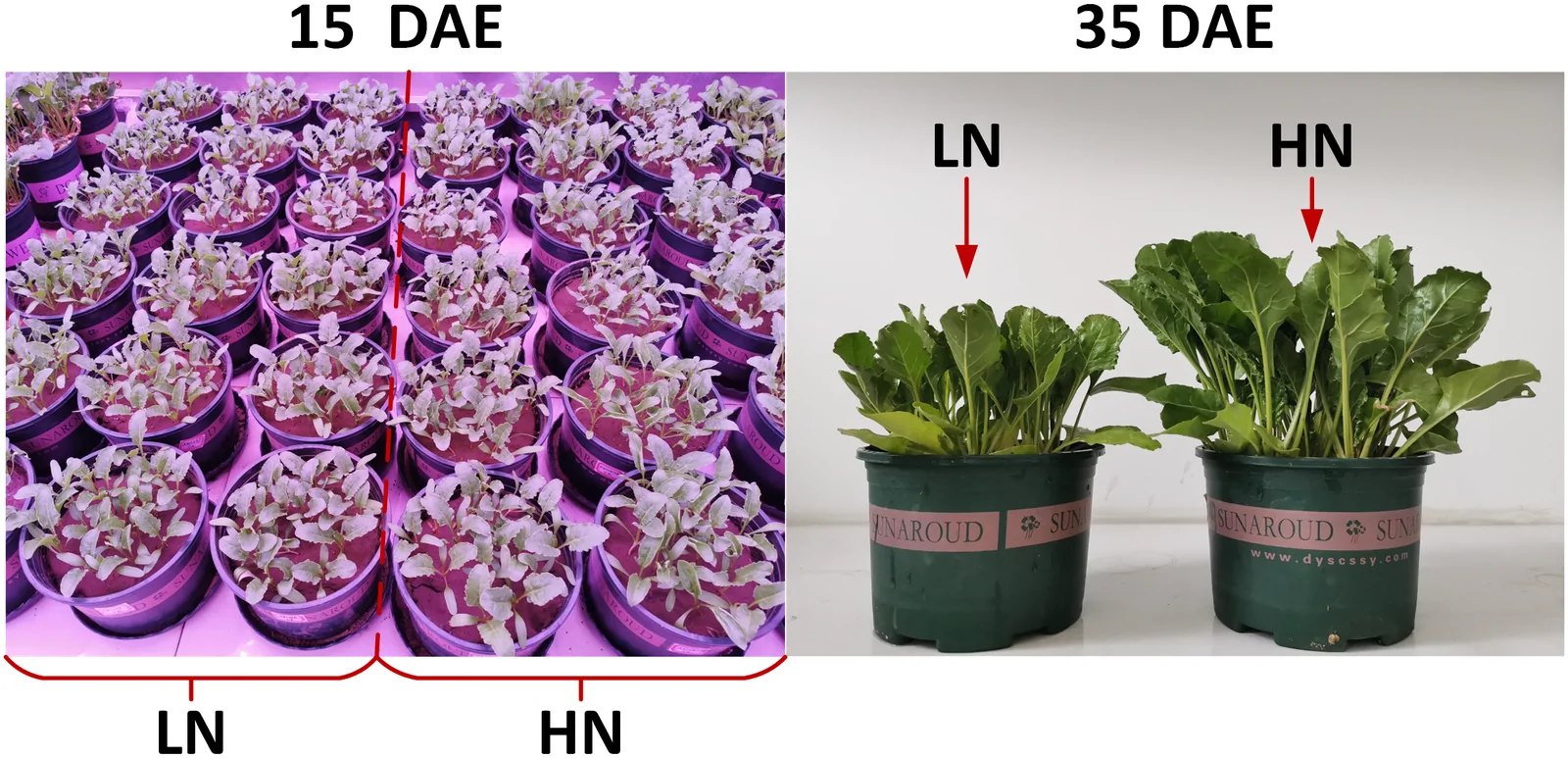
Phenotypic comparison of sugar beet seedlings under HN and LN treatments at 15 and 35 DAE.

### 3.2 Metabolomic analysis of sugar beet under different nitrogen levels

PCA was first performed to evaluate the overall quality and reproducibility of the metabolomic data. In the PCA score plots, quality control (QC) samples clustered tightly near the center, supporting the stability and reliability of the analytical system throughout the experiment. Clear separation between HN and LN groups was observed in both leaf (**Figure 1A**) and root (**Figure 1B**) tissues. In leaves, PC1 and PC2 explained 20.0% and 12.6% of the total variance, respectively. In roots, the corresponding values were 23.5% and 14.1%. The HN group (red circles) and LN group (blue triangles) exhibited distinct clustering patterns in both tissues, confirming that nitrogen supply reshaped the metabolite profiles of sugar beet seedlings.

To further maximize inter-group differences, OPLS-DA modeling was performed on the metabolomic data (**Supplementary Figure S1**). OPLS-DA score plots showed clear separation between HN and LN groups in both leaves and roots. Model reliability was evaluated through 200 permutation tests; all models yielded Q² values greater than 0.5, and the permuted R² and Q² values were lower than those of the original models (**Supplementary Figure S1**), ruling out overfitting and supporting the authenticity of inter-group metabolic differences.

DAMs were identified based on VIP values calculated from the OPLS-DA model, combined with the criteria of VIP > 1.0, FC > 1.2 or FC < 0.833, and nominal *P* < 0.05 (**Supplementary Figure S1**). A sensitivity analysis using Benjamini-Hochberg FDR correction showed that of the 148 leaf DAMs, 17 remained significant at FDR < 0.05; of the 261 root DAMs, 260 remained significant at FDR < 0.05. The four nitrogen metabolism-related DAMs discussed in detail (L-glutamate, L-aspartate, O-phospho-L-serine, and D-erythrose-4-phosphate) remained significant at FDR < 0.05 in at least one ionization mode, supporting their inclusion in downstream correlation analyses. In the leaf comparison (HNVL1 vs. LNVL1), 80 DAMs were identified in positive ion mode (29 upregulated and 51 downregulated under HN)(**Figure 3A**), and 68 DAMs in negative ion mode (41 upregulated and 27 downregulated)(**Figure 3B**). In roots (HNVR1 vs. LNVR1), more DAMs were detected: 132 in positive ion mode (71 upregulated and 61 downregulated)(**Figure 3C**), and 129 in negative ion mode (103upregulated and 26 downregulated) (**Figure 3D**). Roots yielded roughly twice as many DAMs as leaves, consistent with their direct exposure to nitrogen in the substrate, though tissue-specific differences in developmental stage may also contribute to this imbalance. It should be noted that positive- and negative-ion mode data were analyzed as separate datasets, and the total DAM counts reported here (148 in leaves and 261 in roots) represent the sum of DAMs identified in each mode.

**Figure 3:**
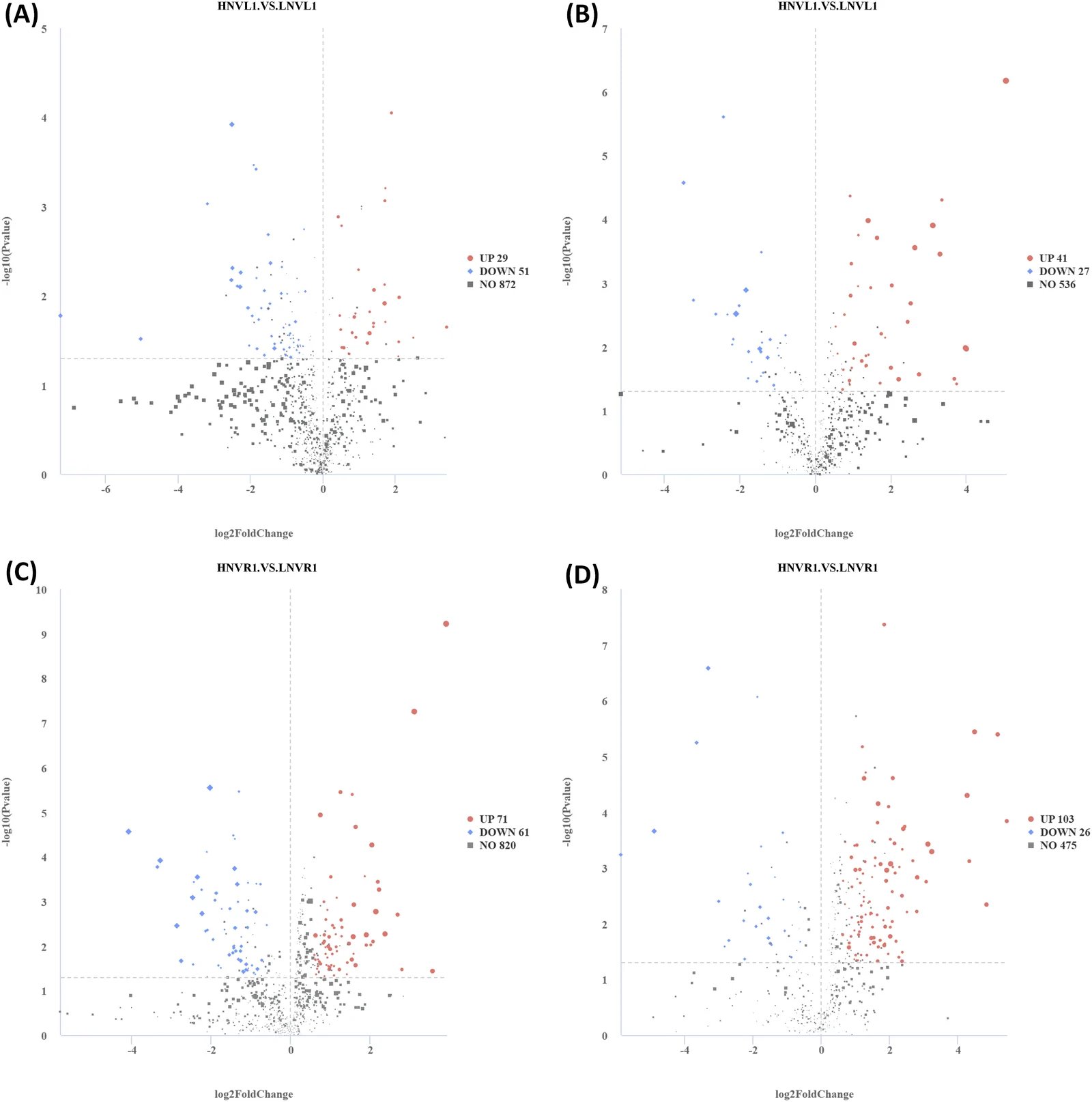
Volcano plots of differentially accumulated metabolites in sugar beet leaves and roots (HNVL1 vs. LNVL1 and HNVR1 vs. LNVR1). **(A, C)** Positive ion mode. **(B, D)** Negative ion mode. Red dots: upregulated metabolites; blue dots: downregulated metabolites; gray dots: metabolites with no significant change.

### 3.3 Transcriptomic analysis of sugar beet under different nitrogen levels

#### 3.3.1 Overview of gene expression and DEG identification

Venn diagram analysis of expressed genes showed that 17, 935 genes were co-expressed in HN and LN leaf samples, with 833 genes expressed exclusively under LN and 745 exclusively under HN (**Figure 4A**). In roots, 18, 585 genes were shared between the two treatments, with 987 being LN-specific and 654 being HN-specific (**Figure 4B**). The higher number of uniquely expressed genes under LN in both tissues implies that lower nitrogen supply activated additional transcriptional programs as part of an adaptive response.

**Figure 4:**
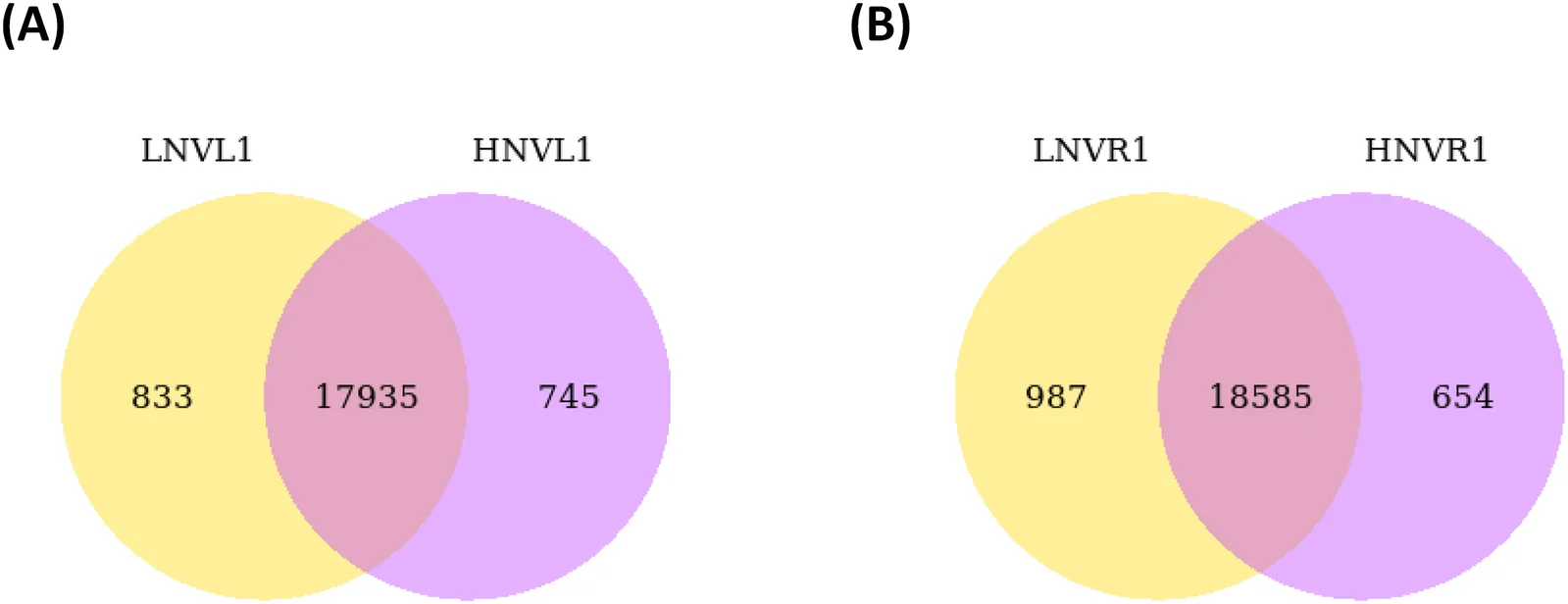
Venn diagrams of co-expressed and uniquely expressed genes between HN and LN treatments. **(A)** Leaves. **(B)** Roots.

Using criteria of |log_2_(fold change)| ≥ 1 and padj ≤ 0.05, a total of 1, 472 DEGs (578 up-regulated and 894 down-regulated in HN) were identified in leaves, and 1, 224 DEGs (485 up-regulated and 739 down-regulated) in roots (**Figure 5**). To screen candidate genes with substantial expression changes, a more stringent threshold of |log_2_(fold change)| ≥ 3 was further applied, yielding 114 DEGs (44 up-regulated and 70 down-regulated) in leaves and 56 DEGs (19 up-regulated and 37 down-regulated) in roots.

**Figure 5:**
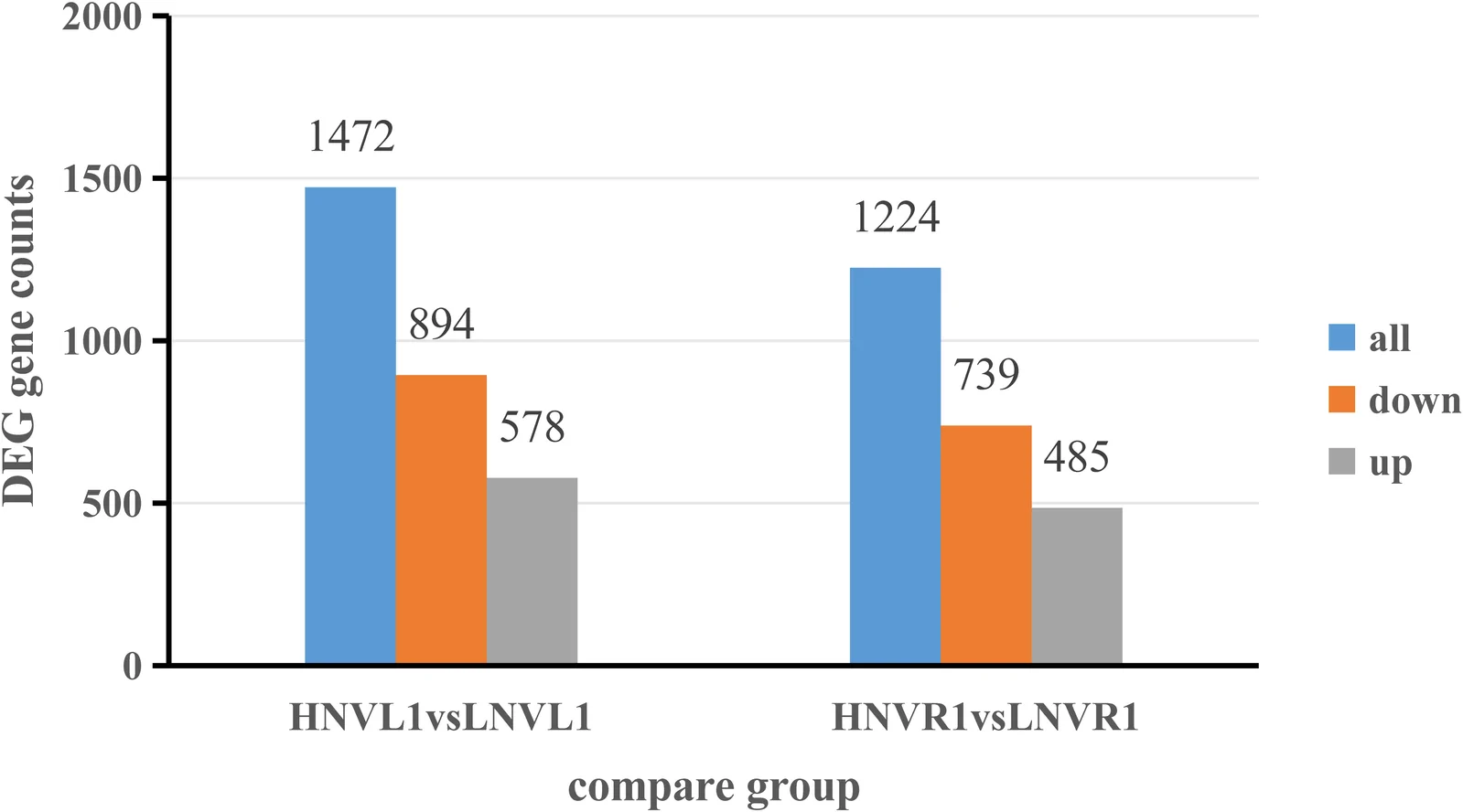
Number of differentially expressed genes (DEGs) identified in leaf and root comparisons (HNVL1 vs. LNVL1 and HNVR1 vs. LNVR1). Blue bars represent total DEGs, red bars represent downregulated DEGs, and gray bars represent upregulated DEGs.

#### 3.3.2 Nitrogen metabolism-related DEGs

Among all identified DEGs, 14 genes related to nitrogen metabolism were recovered from sugar beet leaves and roots (**Table 2**; **Figures 6A, B**). Seven DEGs were found in leaves, of which five were up-regulated and two were down-regulated under HN. Eight DEGs were found in roots, all of which were down-regulated under HN. One DEG, *IMABv06g017432* (encoding high-affinity nitrate transporter 2.5), was present in both tissues.

**Table 2:** Differentially expressed genes related to nitrogen metabolism in sugar beet seedlings.

No.	Gene ID	Gene description	Tissue	direction of expression	chromosome	gene_start	gene_end	gene_strand	GO	KEGG	Gene_length	log2FC	padj	FPKM (HN)	FPKM (LN)
1	*IMABv03g012146*	Glutamate synthase 1 (GLT1)	Leaf	up	GWHAMMD00000003	79162687	79174338	–	dicarboxylic acid metabolic process;alpha-amino acid metabolic process	Nitrogen metabolism;Alanine, aspartate and glutamate metabolism;Biosynthesis of amino acids;Biosynthesis of amino acids	7230	1.13	0.00	6824.53	3120.75
2	*IMABv08g029213*	Carbonic anhydrase	Leaf	up	GWHAMMD00000008	62615088	62622725	+	lyase activity;’hydro-lyase activity	Nitrogen metabolism	1172	1.20	0.00	23181.11	10073.48
3	*IMABv01g023663*	Nitrate reductase (NIA)	Leaf	up	GWHAMMD00000001	730580	737612	–	oxoacid metabolic process;organic acid metabolic process;small molecule metabolic process	Nitrogen metabolism	3269	1.68	0.00	20063.34	6267.45
4	*IMABv04g005591*	Ferredoxin-nitrite reductase (NIR)	Leaf	up	GWHAMMD00000004	19114694	19117790	+	heme binding;tetrapyrrole binding;iron-sulfur cluster binding;metal cluster binding	Nitrogen metabolism	1915	1.12	0.01	2345.16	1078.99
5	*IMABv04g005579*	Ferredoxin-nitrite reductase (NIR)	Leaf	up	GWHAMMD00000004	18859073	18862142	+	iron-sulfur cluster binding;metal cluster binding;heme binding;tetrapyrrole binding	Nitrogen metabolism	2123	1.10	0.02	2617.61	1222.55
6	*IMABv06g017433*	High-affinity nitrate transporter 2.5 (NRT2.5)	Leaf	down	GWHAMMD00000006	13377808	13379754	–	–	Nitrogen metabolism	1620	-5.20	0.00	0.51	18.90
7	*IMABv06g017432*	High-affinity nitrate transporter 2.5 (NRT2.5)	Leaf/Root	down	GWHAMMD00000006	13374425	13375814	–	–	Nitrogen metabolism	1390	-6.56	0.00	0.47	47.97
8	*IMABv09g021027*	Glutamine synthetase nodule isozyme	Root	down	GWHAMMD00000009	20660898	20670760	+	glutamine family amino acid metabolic process;glutamine family amino acid biosynthetic process;alpha-amino acid metabolic process;alpha-amino acid biosynthetic process;cellular amino acid biosynthetic process;organic acid biosynthetic process;carboxylic acid biosynthetic process;cellular amino acid metabolic process;small molecule biosynthetic process;carboxylic acid metabolic process;oxoacid metabolic process;organic acid metabolic process;small molecule metabolic process;ligase activity, forming carbon-nitrogen bonds;ligase activity	Nitrogen metabolism;Arginine biosynthesis;Alanine, aspartate and glutamate metabolism;Biosynthesis of amino acids	1300	-6.56	0.00	23.23	2189.60
9	*IMABv06g017450*	High-affinity nitrate transporter 2.5 (NRT2.5)	Root	down	GWHAMMD00000006	13644815	13648479	–	–	Nitrogen metabolism	1794	-3.71	0.00	14.38	188.55
10	*IMABv07g015769*	High-affinity nitrate transporter 2.1 (NRT2.1)	Root	down	GWHAMMD00000007	75838346	75844394	+	–	Nitrogen metabolism	1931	-3.49	0.00	67.96	766.41
11	*IMABvctg032743*	High-affinity nitrate transporter 2.5 (NRT2.5)	Root	down	GWHAMMD00000141	9556	12239	+	–	Nitrogen metabolism	1809	-2.44	0.00	12.43	67.49
12	*IMABv06g017443*	High-affinity nitrate transporter 2.5 (NRT2.5)	Root	down	GWHAMMD00000006	13557528	13558887	–	–	Nitrogen metabolism	1360	-2.50	0.01	17.89	101.37
13	*IMABv06g017440*	High-affinity nitrate transporter 2.5 (NRT2.5)	Root	down	GWHAMMD00000006	13512940	13514347	–	–	Nitrogen metabolism	1408	-2.90	0.01	2.24	16.77
14	*IMABv06g019714*	Formamidase	Root	down	GWHAMMD00000006	78241303	78244351	+	hydrolase activity, acting on carbon-nitrogen (but not peptide) bonds;hydrolase activity, acting on carbon-nitrogen (but not peptide) bonds, in linear amides;	Nitrogen metabolism;Cyanoamino acid metabolism;Carbon metabolism;Glyoxylate and dicarboxylate metabolism	1492	-1.69	0.03	18.13	58.24

The table lists gene ID, gene description, tissue, direction of expression, chromosome, gene_strand, GO, KEGG, gene length, log2 fold change (log2FC), adjusted P (padj), and FPKM values under HN and LN treatments, allowing comparison of both annotation and expression abundance for each candidate nitrogen metabolism-related DEG.

**Figure 6:**
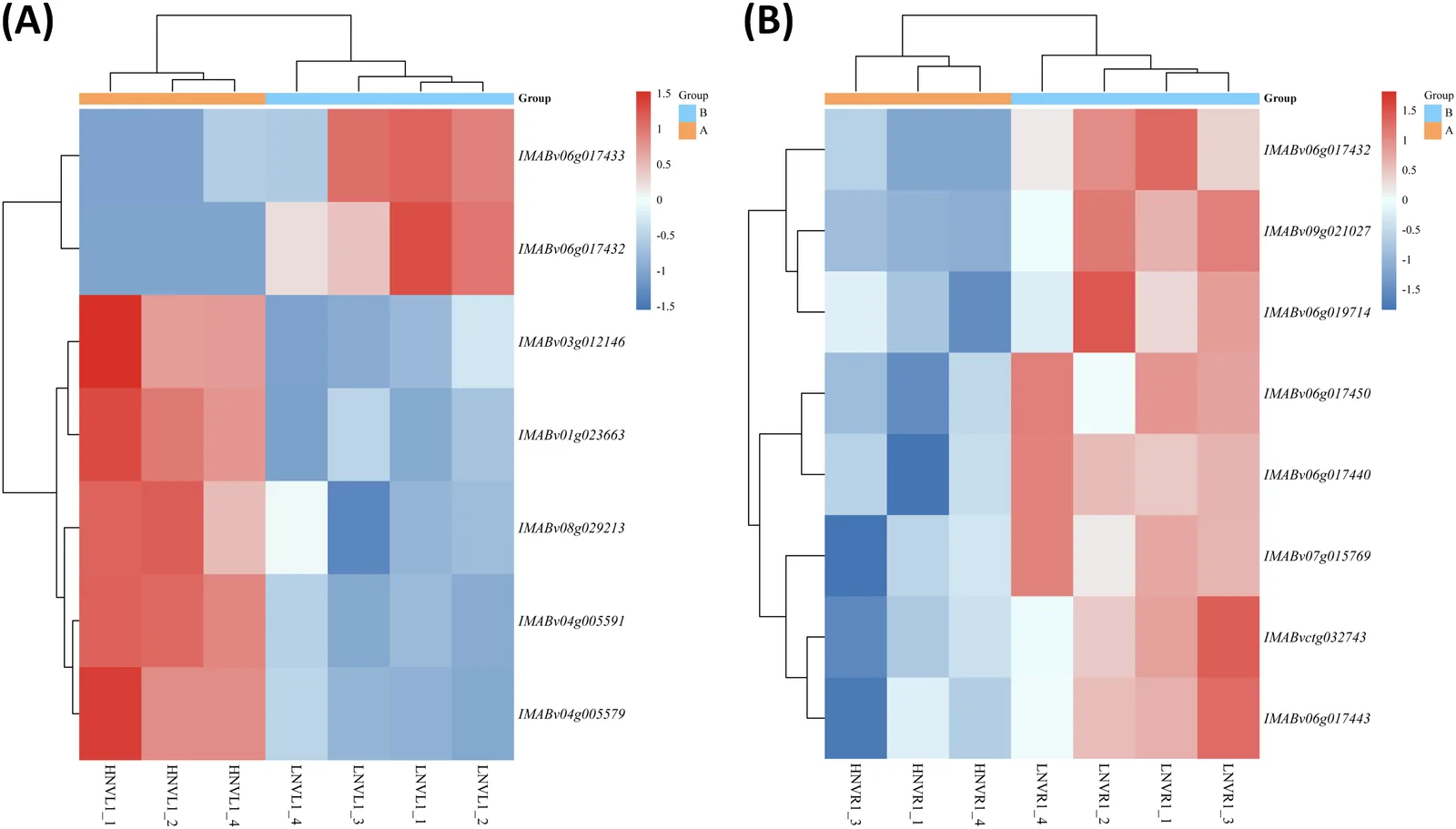
**(A, B)** represent the clustering heatmap of differentially expressed genes related to nitrogen metabolism in leaves and roots respectively. The sample HNVL1_3 is omitted from **Figure 6A** due to expression patterns that are inconsistent with the other HNVL1 samples.

The identified nitrogen metabolism-related DEGs encode enzymes and transporters involved in the nitrogen assimilation pathway, including glutamate synthase (*GLT1*, *IMABv03g012146*), nitrate reductase (*NIA*, *IMABv01g023663*), ferredoxin-nitrite reductase (*NIR*, *IMABv04g005591* and *IMABv04g005579*), high-affinity nitrate transporters (*NRT2.5* and *NRT2.1*), carbonic anhydrase (*IMABv08g029213*), glutamine synthetase nodule isozyme (*IMABv09g021027*), and formamidase (*IMABv06g019714*).

Expression analysis showed tissue-specific patterns. In leaves, *IMABv03g012146* (*GLT1*), *IMABv04g005591* (*NIR*), and *IMABv04g005579* (*NIR*) showed higher expression under HN (padj < 0.05), reflecting enhanced nitrate reduction and glutamate biosynthesis. *IMABv06g017432* (*NRT2.5*) and *IMABv06g017433* (*NRT2.5*) were up-regulated under LN in both leaves and roots, matching the expected compensatory activation of high-affinity nitrate transport under nitrogen limitation. In roots, all eight DEGs encoding nitrate transporters were down-regulated under HN, a pattern consistent with reduced demand for high-affinity nitrogen uptake when nitrogen supply is increased.

#### 3.3.3 qRT-PCR validation of DEGs

qRT-PCR was employed to validate the expression patterns of the 14 nitrogen metabolism-related DEGs identified by RNA-seq (**Figure 7**). In leaves, six DEGs (*IMABv04g005591*, *IMABv08g029213*, *IMABv03g012146*, *IMABv01g023663*, *IMABv04g005579*, and *IMABv06g019714*) showed higher expression under HN than LN (*P* < 0.05 or *P* < 0.01), in agreement with the RNA-seq results. Two DEGs (*IMABv09g021027* and *IMABv06g017433*) were more highly expressed under LN in leaves.

**Figure 7:**
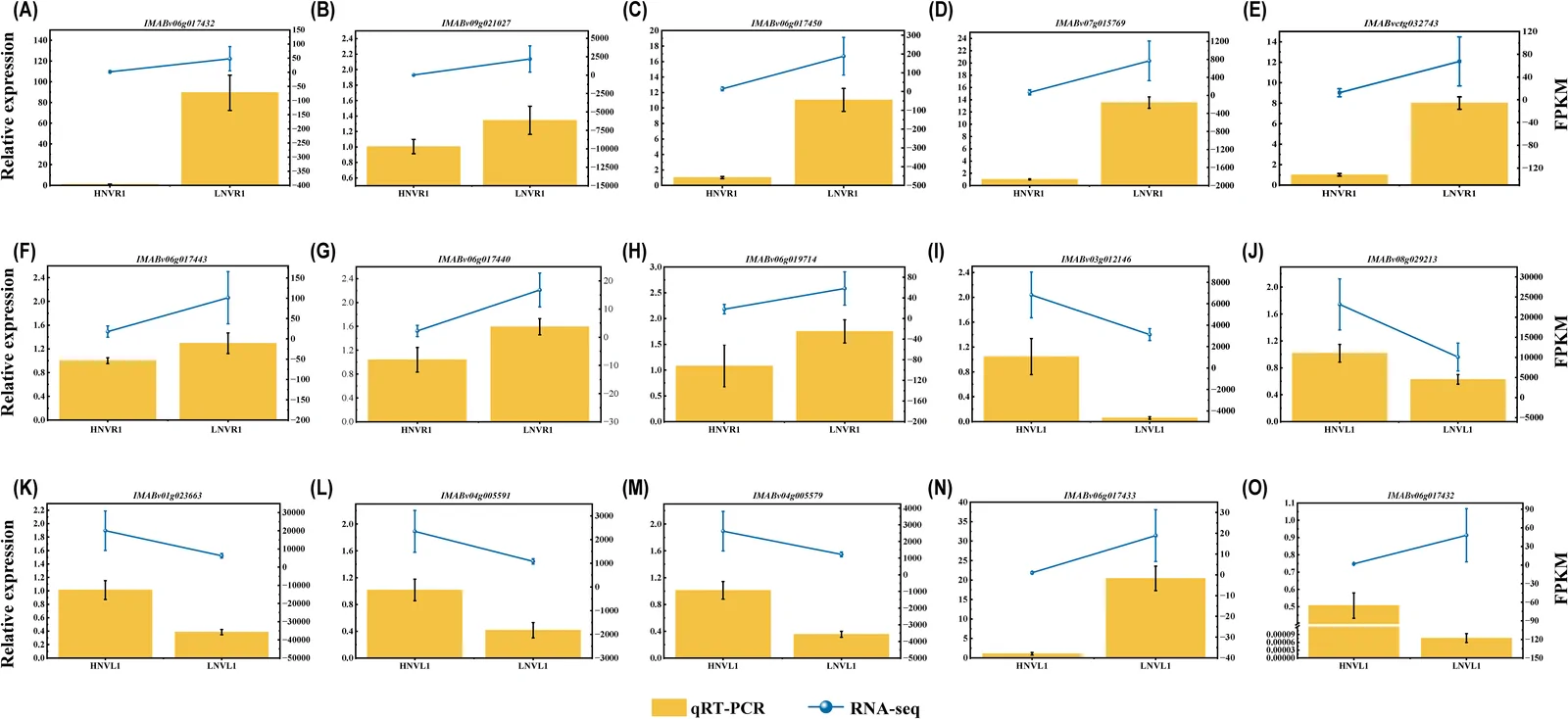
Validation of fifteen nitrogen metabolism-related DEGs by qRT-PCR. **(A)** *IMABv06g017432*, **(B)** *IMABv09g021027*, **(C)** *IMABv06g017450*, **(D)** *IMABv07g015769*, **(E)** *IMABvctg032743*, **(F)** *IMABv06g017443*, **(G)** *IMABv06g017440*, **(H)** *IMABv06g019714*, **(I)** *IMABv03g012146*, **(J)** *IMABv08g029213*, **(K)** *IMABv01g023663*, **(L)** *IMABv04g005591*, **(M)** *IMABv04g005579*, **(N)** *IMABv06g017433*, and **(O)** *IMABv06g017432*. The relative expression levels determined by qRT-PCR (yellow bars, calculated using the (2^-ΔΔCt^ method) are shown together with the normalized expression values (FPKM) from RNA-seq (blue line). Error bars represent the standard deviation of three biological replicates.

In roots, *IMABv03g012146* and *IMABv04g005579* were up-regulated under HN (*P* < 0.01). Three nitrate transporter genes, *IMABv06g017450*, *IMABv07g015769*, and *IMABv06g017432*, showed higher expression under LN, matching the expected compensatory upregulation of high-affinity nitrate transporters under lower nitrogen supply. Overall, the qRT-PCR results agreed with the RNA-seq data, supporting the reliability of the transcriptomic analysis.

### 3.4 Integrated transcriptome and metabolome analysis

#### 3.4.1 KEGG co-enrichment analysis

Joint analysis of transcriptomic and metabolomic data was performed to identify pathways co-enriched by DEGs and DAMs (**Supplementary Table S1**, **Figures 8A, B**). In leaves, KEGG annotation results indicated that the amino acid biosynthesis pathway was co-enriched in both the transcriptome and metabolome, comprising DEG *IMABv03g012146* (glutamate synthase) and the differentially accumulated metabolites O-phospho-L-serine, D-erythrose-4-phosphate, and N-acetylornithine.

**Figure 8:**
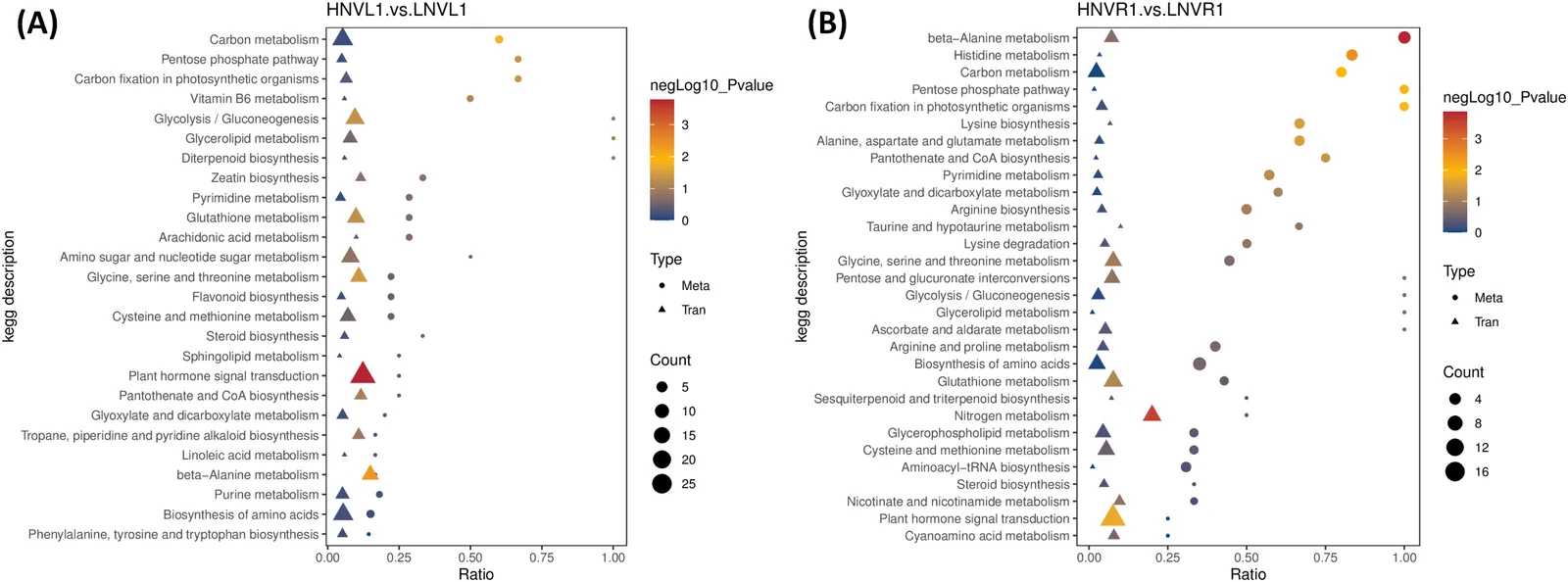
KEGG enrichment bubble plots of DEGs and differentially accumulated metabolites. **(A)** Sugar beet leaves, positive ion mode. **(B)** Sugar beet roots, positive ion mode. The x-axis represents the proportion of enriched differential metabolites or genes relative to the total annotated number in each pathway. The y-axis represents co-enriched KEGG pathways. Circles: metabolites; triangles: transcripts.

In roots, KEGG annotation results showed broader co-enrichment patterns (**Supplementary Table S1**, **Figure 8B**). In the nitrogen metabolism pathway, eight DEGs encoding nitrate transporters and nitrogen assimilation enzymes were co-enriched with L-glutamate, linking nitrogen uptake directly to glutamate synthesis in roots at both the transcript and metabolite levels. In the alanine-aspartate-glutamate metabolism pathway, the glutamine synthetase gene (*IMABv09g021027*) was co-enriched with four metabolites: alpha-ketoglutarate, L-aspartate, L-glutamate, and gamma-aminobutyric acid (GABA), implying that this pathway bridges nitrogen assimilation and carbon skeleton metabolism. GABA, as both a metabolite of the GABA shunt and a signaling molecule, may play a dual role in linking nitrogen status to stress adaptation and growth regulation (Ramesh et al., 2015; Carillo, 2018). The arginine biosynthesis and amino acid biosynthesis pathways were also simultaneously enriched by DEGs and multiple amino acid DAMs, pointing to broad amino acid metabolic reprogramming in roots in response to nitrogen-level changes. The specific genes and metabolites involved in each co-enriched pathway are detailed in **Supplementary Table S1**.

#### 3.4.2 Nitrogen metabolism pathway reconstruction

Based on KEGG analysis, the involvement of the 14 nitrogen metabolism-related DEGs in sugar beet-specific nitrogen metabolism pathways was mapped and visualized (**Figure 9**). In both leaves and roots, extracellular nitrate is transported into cells through high-affinity nitrate transporters (NRT2.5 and NRT2.1) encoded by multiple DEGs. Intracellular nitrate is then converted to nitrite by nitrate reductase (NIA, *IMABv01g023663*), or extracellular nitrite is directly transported into cells. Nitrite is further reduced to ammonium by ferredoxin-nitrite reductase (NIR, *IMABv04g005591* and *IMABv04g005579*).

**Figure 9:**
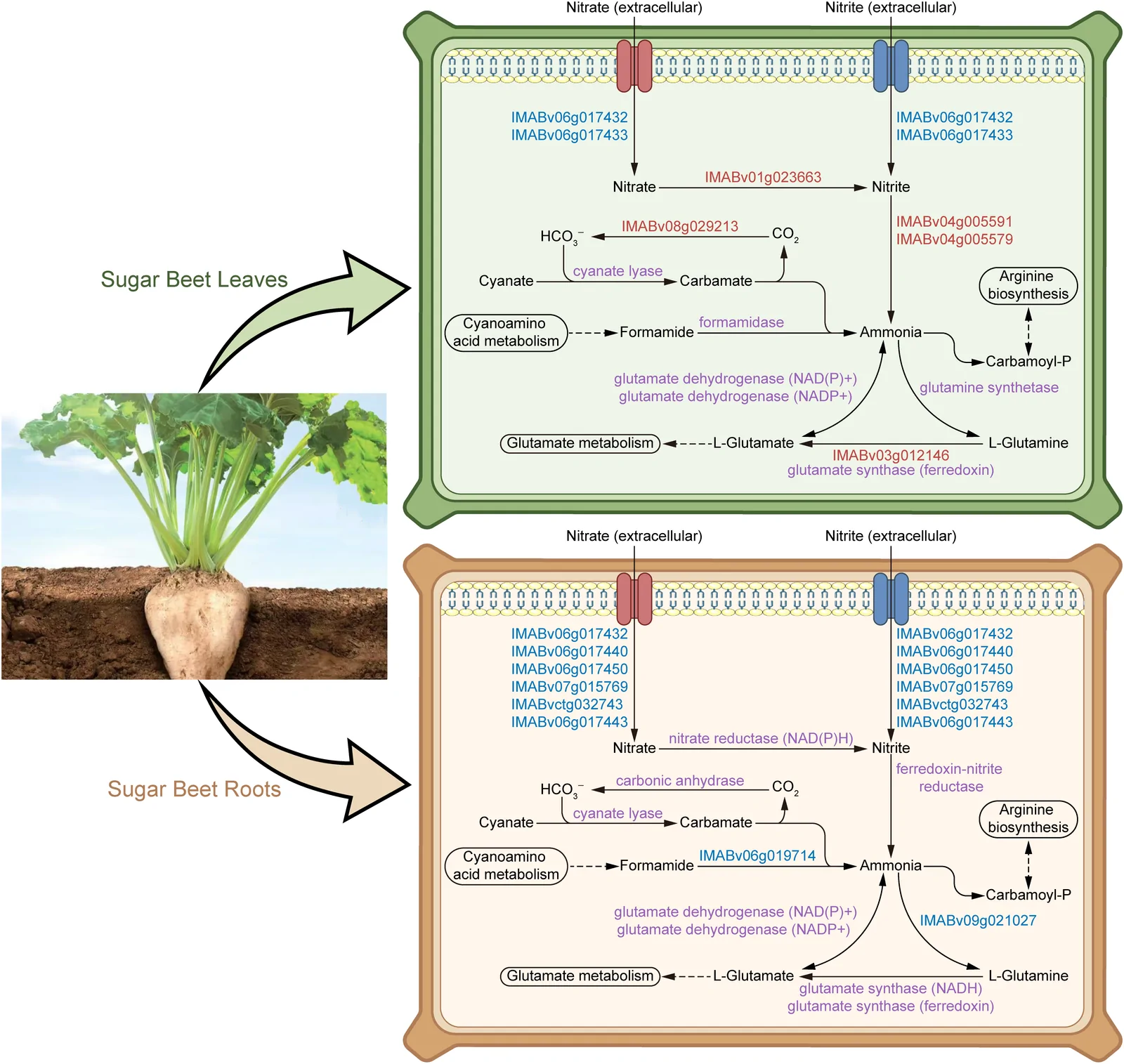
KEGG pathway reconstruction of nitrogen metabolism in sugar beet seedlings under different nitrogen levels. The red and blue fonts represent genes, with red representing genes with upregulated expression levels, blue representing genes with downregulated expression levels, and purple representing enzymes involved in N metabolism pathways.

The downstream fate of ammonium diverges into multiple pathways. A portion of ammonium is converted to carbamoyl phosphate, entering the arginine biosynthesis pathway. Another portion is assimilated into glutamine by glutamine synthetase (*IMABv09g021027*), and glutamine is subsequently converted to glutamate by glutamate synthase (GLT1, *IMABv03g012146*), entering the glutamate metabolism pathway. Carbonic anhydrase (*IMABv08g029213*) catalyzes the conversion of cyanate to carbamate, while formamidase (*IMABv06g019714*) mediates the conversion of formamide to ammonium in the cyanoamino acid metabolism pathway.

Of the 14 DEGs, five involved in nitrogen assimilation in leaves were up-regulated under HN, participating in the stepwise conversion of nitrate to nitrite, ammonium, glutamine, and glutamate. By contrast, all eight root-specific DEGs (predominantly encoding nitrate transporters) were down-regulated under HN, in line with reduced demand for high-affinity nitrogen uptake when nitrogen supply is abundant, collectively reconstructing the nitrogen-responsive metabolic network in sugar beet seedlings.

#### 3.4.3 Correlation between nitrogen metabolism-related DEGs and DAMs

To examine the association between gene expression and metabolite accumulation, Pearson correlation analysis was performed between the 14 nitrogen metabolism-related DEGs and four DAMs (L-glutamate, L-aspartate, O-phospho-L-serine, and D-erythrose-4-phosphate) (**Figure 10**).

**Figure 10:**
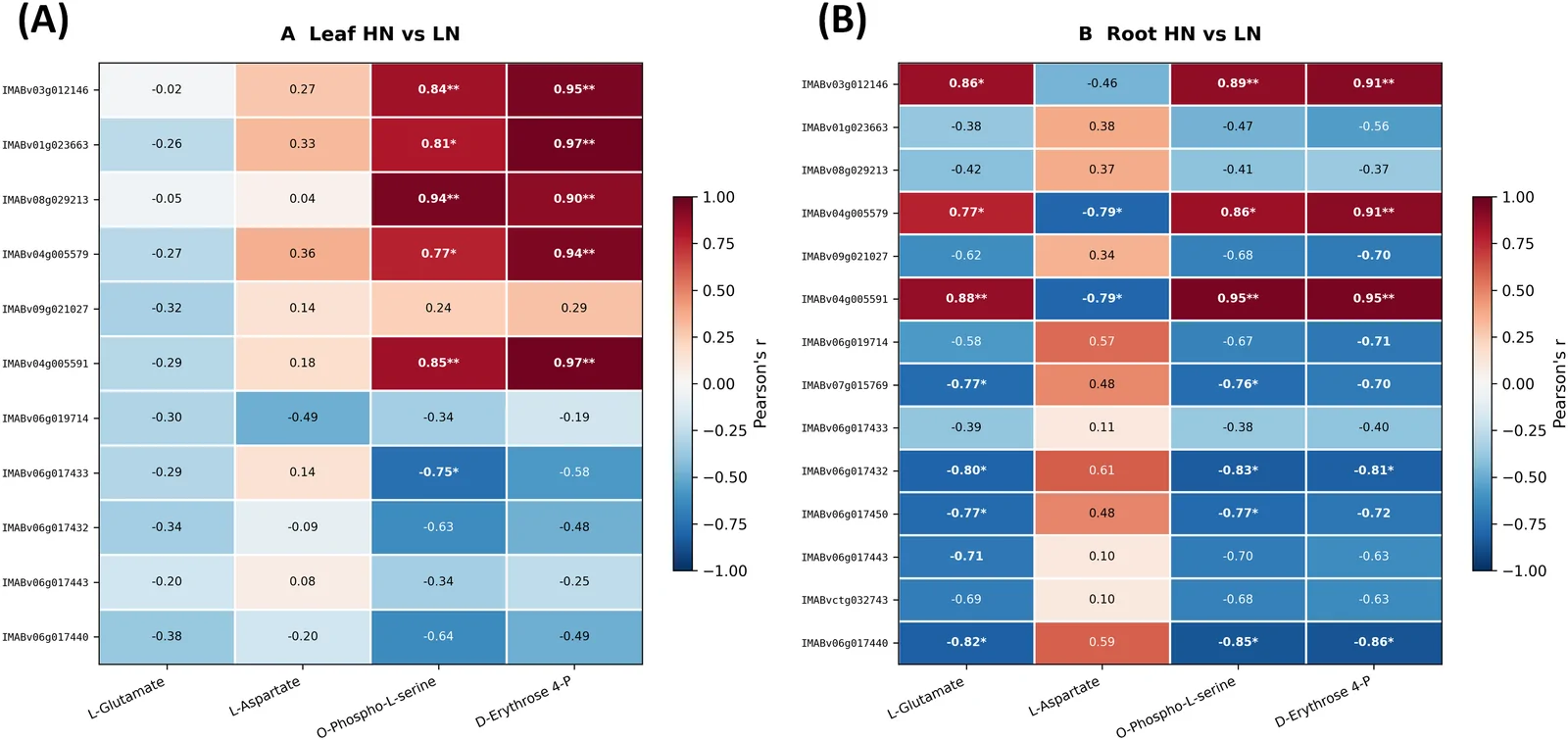
Pearson correlation heatmaps between nitrogen metabolism-related DEGs and key DAMs. **(A)** Leaf tissue (11 expressed genes). **(B)** Root tissue (14 genes). The color scale represents the Pearson correlation coefficient (r); red indicates positive correlation and blue indicates negative correlation. *P < 0.05; **P < 0.01.

In leaves (**Figure 10A**), five DEGs encoding nitrogen assimilation enzymes, namely *IMABv03g012146* (*GLT1*), *IMABv01g023663* (*NIA*), *IMABv08g029213* (*CA*), *IMABv04g005591* (*NIR*), and *IMABv04g005579* (*NIR*), showed strong positive correlations with O-phospho-L-serine (r = 0.77 to 0.94) and D-erythrose-4-phosphate (r = 0.90 to 0.97). However, correlations with L-glutamate (r = -0.27 to -0.02) and L-aspartate (r = 0.04 to 0.36) were weak. Three *NRT2* family genes, *IMABv06g017433*, *IMABv06g017432*, and *IMABv06g017440*, showed negative correlations with O-phospho-L-serine (r = -0.75 to -0.63) and D-erythrose-4-phosphate (r = -0.58 to -0.48). Three additional DEGs (*IMABv06g017450*, *IMABv07g015769*, and *IMABvctg032743*) were barely expressed in leaves and were therefore excluded from the leaf correlation analysis.

In roots (**Figure 10B**), six NRT2 family genes (*IMABv06g017432*, *IMABv06g017440*, *IMABv07g015769*, *IMABv06g017450*, *IMABv06g017443*, and *IMABvctg032743*) exhibited negative correlation trends with L-glutamate (r = -0.82 to -0.69), as well as with O-phospho-L-serine (r = -0.86 to -0.68) and D-erythrose-4-phosphate (r = -0.86 to -0.63). *IMABv09g021027* (glutamine synthetase, GS) showed a negative correlation with L-glutamate (r = -0.62) and a weak positive correlation with L-aspartate (r = 0.35). Three nitrogen assimilation genes (*IMABv03g012146*, *IMABv04g005591*, and *IMABv04g005579*) showed positive correlations with L-glutamate (r = 0.77 to 0.88) and D-erythrose-4-phosphate (r = 0.91 to 0.95) in roots.

## 4 Discussion

### 4.1 Nitrogen supply promotes above-ground growth and chlorophyll accumulation

Sugar beet growth and yield depend heavily on nitrogen supply. Nitrogen is required for the synthesis of proteins, nucleic acids, and other macromolecules (Zayed et al., 2023), and its effects on growth and photosynthesis have been documented in numerous crops, including rice (Zhao et al., 2023), maize (Wang et al., 2020; Meng et al., 2024), potato, and tomato (Sun et al., 2023). In sugar beet, nitrogen supply affects leaf area, photosynthetic potential, and root cell size (Li et al., 2023a), as well as yield and sugar content (Li et al., 2024). Our data extend these observations: nitrogen fertilization increased plant height, fresh weight, and leaf chlorophyll content (SPAD) at 35 DAE (**Table 1**), though no measurable differences appeared at 15 DAE. This lag indicates that the effect of nitrogen supply on seedling growth was cumulative. The SPAD response provides physiological evidence that nitrogen supply affected leaf chlorophyll status, supporting the effectiveness of the treatment contrast. However, tissue total nitrogen, nitrate concentration, nitrate reductase activity, and nitrogen uptake/use efficiency were not measured in the present sample set. Therefore, the omics results should be interpreted as molecular and metabolic associations with the treatment contrast rather than as direct measurements of nitrogen uptake or nitrogen-use efficiency.

### 4.2 Transcriptomic responses indicate tissue-specific regulation of nitrogen metabolism genes

Our transcriptomic analysis identified 1, 472 DEGs in leaves and 1, 224 DEGs in roots (**Figure 5**), of which 14 were directly related to nitrogen metabolism, encoding enzymes and transporters involved in nitrate uptake, reduction, and amino acid biosynthesis (**Table 2**). These 14 DEGs fell into two contrasting groups by tissue. In leaves, five up-regulated DEGs (*IMABv03g012146*, *IMABv04g005591*, *IMABv04g005579*, *IMABv01g023663*, and *IMABv08g029213*) encode enzymes catalyzing the stepwise conversion of nitrate to nitrite, ammonium, and finally glutamate, pointing to activated nitrogen assimilation when nitrogen supply is adequate. In roots, the pattern reversed: all eight root-specific DEGs, most encoding high-affinity nitrate transporters (NRT2.5 and NRT2.1), were down-regulated under HN. A similar compensatory up-regulation of NRT2 genes under nitrogen limitation has been reported in barley (Guo et al., 2020), wheat (Deng et al., 2023), and other species (Li et al., 2023b; Liu et al., 2020a), and in sugar beet roots this response likely serves to scavenge the limited nitrogen available in the substrate (Li et al., 2023a).

This “root uptake, leaf assimilation” model is physiologically plausible: roots, as the primary barrier for nitrogen absorption, preferentially activate the high-affinity transport system under nitrogen limitation to maintain nitrogen uptake; leaves, as the primary site for nitrogen assimilation and photosynthesis, up-regulate assimilation enzymes under high nitrogen supply to efficiently use absorbed inorganic nitrogen. This spatial model may be coordinated by systemic nitrogen signaling. In *Arabidopsis*, NLP (NIN-like protein) transcription factors have been shown to integrate nitrate signals and regulate the expression of downstream nitrogen metabolism genes (Konishi and Yanagisawa, 2013; Marchive et al., 2013). Recent studies have further demonstrated that NLP7 functions as a primary nitrate sensor through a CPK-mediated signaling cascade (Liu et al., 2017). Whether sugar beet uses a similar NLP-mediated long-distance signaling mechanism remains to be tested, but the clear tissue-level separation we observed is at least compatible with such a model.

Among the 14 nitrogen metabolism DEGs identified here, seven belong to the NRT2 family, and five of these (*IMABv06g017432*, *IMABv06g017433*, *IMABv06g017443*, *IMABv06g017440*, and *IMABv06g017450*) are located on chromosome 6, raising the possibility that these genes arose through tandem duplication. We did not initially expect such strong clustering; the concentration of five NRT2 paralogs within a single chromosomal region points to a localized expansion event that may have given sugar beet finer-grained control over nitrogen transport under nitrogen-limited conditions. Comparable gene family expansions have been reported in other crops adapting to nutritional stress (Guo et al., 2020; Deng et al., 2023). qRT-PCR validation supported these expression patterns (**Figure 7**), lending confidence to the transcriptomic data.

### 4.3 Metabolomic changes reflect adaptive metabolic reprogramming

Metabolomic analysis showed that roots had a broader metabolic response than leaves, with 261 nominally significant candidate metabolites identified in roots compared with 148 in leaves. These DAMs were defined using nominal *P* < 0.05 without FDR correction (see Methods); while the sensitivity analysis indicates that a subset remains significant at FDR < 0.10, the full DAM sets should be interpreted as exploratory candidates rather than confirmed differential metabolites. Under the LN treatment, sugar beet roots accumulated higher levels of certain amino acids and polyamines, possibly as part of a stress-response program aimed at maintaining basic physiological functions (Hildebrandt et al., 2015; Chang et al., 2024). Under high nitrogen conditions, the increased availability of inorganic nitrogen likely drives greater synthesis of proteins, nucleic acids, and growth-associated secondary metabolites. We identified four differentially accumulated metabolites central to nitrogen metabolism: L-glutamate, L-aspartate, O-phospho-L-serine, and D-erythrose-4-phosphate. L-glutamate and L-aspartate are core products of the GS/GOGAT cycle and aspartate aminotransferase reactions, serving as amino donors for numerous other amino acids and nitrogen-containing compounds. O-phospho-L-serine is an intermediate of serine biosynthesis via the phosphorylated pathway, and its accumulation changes may track metabolic flux redistribution between nitrogen assimilation and the photorespiratory pathway (Perez-Delgado et al., 2016; Igamberdiev and Kleczkowski, 2018). D-erythrose-4-phosphate links the pentose phosphate pathway to aromatic amino acid biosynthesis (the shikimate pathway); its differential accumulation implies that nitrogen-level changes affect not just primary nitrogen metabolism but also, indirectly, secondary metabolite precursors through carbon skeleton supply. Similar carbon-nitrogen crosstalk has been observed in rice (Xin et al., 2019a, b).

### 4.4 Integrated analysis links gene expression with metabolite accumulation

KEGG co-enrichment analysis of transcriptomic and metabolomic data linked specific gene expression changes to metabolite shifts in sugar beet under different nitrogen conditions. In roots, multiple nitrogen metabolism pathways were co-enriched by DEGs and DAMs, including nitrogen metabolism, alanine/aspartate/glutamate metabolism, arginine biosynthesis, and amino acid biosynthesis (**Figure 8**). The co-occurrence of eight DEGs and L-glutamate in the nitrogen metabolism pathway places glutamate metabolism at the center of nitrogen assimilation in sugar beet roots. Li et al. (2023a) similarly reported glutamate dehydrogenase B (*GLUDB*) as a down-regulated gene bridging carbon, nitrogen, and glutamate metabolism, which aligns with the pattern we observe here.

Changes in DEG expression and metabolite levels likely influence each other. Up-regulation of nitrogen assimilation genes can increase amino acid pools (Perchlik and Tegeder, 2017), while metabolites such as glutamate and glutamine themselves act as signaling molecules that feed back to gene regulation (Baslam et al., 2020; Xin et al., 2019a). The reconstructed nitrogen metabolism pathway (**Figure 9**) offers a molecular framework for these coordinated gene-metabolite interactions in sugar beet leaves and roots.

Pearson correlation analysis further quantified the relationships between nitrogen metabolism-related gene expression and metabolite accumulation (**Figure 10**). In leaves, five nitrogen assimilation genes (encoding GLT1, NIA, CA, and NIR) showed strong positive correlation trends with O-phospho-L-serine and D-erythrose-4-phosphate (r = 0.77 to 0.97), but weak associations with L-glutamate and L-aspartate. This pattern is compatible with the interpretation that these genes, when up-regulated under HN, promote the biosynthesis of amino acid precursors such as O-phospho-L-serine rather than directly increasing primary amino acid pools. The opposite correlation patterns of *NRT2* family genes in both tissues reinforce the interpretation that these transporters are coupled to specific metabolic outputs in a tissue-dependent way. In roots, the negative correlation trends between *NRT2* family genes and L-glutamate, O-phospho-L-serine, and D-erythrose-4-phosphate suggest that the compensatory upregulation of nitrate transporters under LN is accompanied by reduced accumulation of these nitrogen-containing metabolites, possibly reflecting the metabolic consequences of nitrogen limitation. Three nitrogen assimilation genes (*IMABv03g012146*, *IMABv04g005591*, and *IMABv04g005579*) showed positive correlations with L-glutamate and D-erythrose-4-phosphate (r = 0.77 to 0.95) in roots, suggesting that although these genes are more highly expressed in leaves, they may also participate in nitrogen assimilation in root tissues. These gene-metabolite associations complement the pathway-level analysis and identify specific candidates for future functional experiments, particularly the NRT2.5-glutamate axis in roots.

### 4.5 Limitations and future perspectives

This study has certain boundaries. We used only two nitrogen levels and lacked intermediate concentrations or a nitrogen-free control. Omics samples were collected at a single time point (30 DAE), without considering the dynamic changes in nitrogen response. In addition, we did not conduct validation of physiological indicators such as nitrate concentration and nitrate reductase activity, nor did we verify the protein levels. Moreover, we did not address the potential ion imbalance issues that may arise from the ratio of nitrate to ammonium nitrogen in the nitrogen fertilizer. We also acknowledge that the transcriptomic-metabolomic integration presented in our research is primarily descriptive: it identifies co-occurring gene and metabolite changes within shared pathways and quantifies pairwise associations, but it does not model cross-omics covariance or infer causal regulatory relationships. The value of this approach lies in generating a candidate list of gene-metabolite pairs for targeted follow-up, rather than in providing a mechanistic model of nitrogen-response regulation. However, the primary purpose of our research was to identify the differentially expressed genes and metabolite changes under the two contrasting nitrogen supply conditions, laying the foundation for subsequent functional validation.

The future research focus will be on conducting functional validation of the identified genes and metabolites related to nitrogen metabolism. The NRT2.5 cluster on chromosome 6 is a promising candidate for CRISPR knockout or overexpression experiments (Chen et al., 2024) to test whether individual paralogs have distinct roles under nitrogen limitation. Proteomic data would fill the gap between transcript and metabolite levels, and field trials would ultimately determine whether the candidate genes identified here have practical relevance for nitrogen-use efficiency (Govindasamy et al., 2023). Our ultimate goal is to provide molecular-level theoretical basis for improving the nitrogen fertilizer utilization efficiency of sugar beets.

## 5 Conclusion

Using combined transcriptomic and metabolomic analyses of sugar beet seedling leaves and roots, we found that nitrogen effects on growth are cumulative, becoming measurable at 35 DAE as increases in plant height, fresh weight, and chlorophyll content. Fourteen nitrogen metabolism-related DEGs showed tissue-specific patterns: leaf DEGs encoded nitrogen assimilation enzymes up-regulated under high nitrogen, whereas root DEGs encoded NRT2 high-affinity transporters up-regulated under low nitrogen. Five NRT2 paralogs clustered on chromosome 6, pointing to a tandem expansion that may have broadened the regulatory range for nitrogen uptake. Four metabolites (L-glutamate, O-phospho-L-serine, D-erythrose-4-phosphate, and L-aspartate) correlated with nitrogen metabolism DEGs, reflecting potential crosstalk between carbon and nitrogen metabolism. Together, these genes and metabolites outline a molecular framework for nitrogen response in sugar beet and provide candidate genes and metabolites for future functional validation aimed at improving nitrogen use efficiency.

## Data Availability

The datasets presented in this study can be found in online repositories. The names of the repository/repositories and accession number(s) can be found in the article/**Supplementary Material**.
